# A case of severe opioid and methamphetamine use disorder in a 14 year old

**DOI:** 10.1186/s13722-024-00487-1

**Published:** 2024-07-19

**Authors:** Nadia Allami, Kristen O’Connor, Sarah M. Bagley

**Affiliations:** 1 Santa Cruz Community Health, 1510 Capitola Road, Santa Cruz, CA 95062 USA; 2 Chobanian & Avedisian School of Medicine and Boston Medical Center, 801 Mass Ave, Boston, MA 02118 USA

## Abstract

We present the case of a 14-year-old who established care at our primary care clinic after hospitalization for unintentional fentanyl overdose. They were diagnosed with severe opioid use disorder (OUD) and stimulant use disorder (StUD) and initiated buprenorphine while inpatient. They were then transitioned to the only known outpatient primary care clinic in her county who was actively providing medications for opioid use disorder (MOUD) in adolescents.

At the first visit, they reported a history of 20 overdoses, struggling with adherence to buprenorphine and continued opioid cravings. An overdose safety plan was reviewed with them and their parent including providing them naloxone kits, fentanyl test strips, and education handout sheets. Due to their significant overdose history and adherence challenges with sublingual buprenorphine, they were started on long-acting injectable buprenorphine (LAIB) with weekly provider visits and urine toxicology screening. In collaboration with the treatment team, they initiated behavioral treatment with contingency management (CM), with incentives for appointment completion, expected urine results, and successful medication administration. Over the next 19 months, and to date, they have increasingly engaged with care and have remained abstinent. LAIB may be an appealing alternative for adolescents with OUD to improve adherence and reduce risk of recurrent use and overdose. Adjunctive treatment with CM may improve retention in MOUD and have the benefit of treating StUD. There is a need for further research to explore innovative, community-based treatment for youth with OUD.

## Introduction

Overdose deaths among adolescents aged 14–18 increased 94% from 2019 to 2020 and then an additional 20% in 2021 [1]. Approximately 75% of all overdose deaths in adolescents are due to fentanyl [1]. Most youth deaths involve an opioid plus another substance such as cocaine, benzodiazepine, and methamphetamine [2]. Medication for opioid use disorder (MOUD) are a potent intervention that improve mortality, reduce risk of nonfatal overdose, and improve retention in care [3]. There are three FDA approved MOUD: buprenorphine, methadone, and naltrexone. Sublingual buprenorphine is approved for ages 16 years and older. Evidence for MOUD among adolescents is limited compared to adults, but studies have found consistent benefit including improved engagement in care, a decrease in risk behaviors, and increased abstinence [3]. Treatment with MOUD is recommended by professional organizations including American Society of Addiction Medicine, American Academy of Pediatrics, Society for Adolescent Health and Medicine, and the American Academy of Child and Adolescent Psychiatry [4–7]. However receipt among adolescents is low and decreasing [8].

Contingency management (CM) is a behavioral approach that provides positive rewards to reinforce positive behaviors (e.g., abstinence from substances) and has been an effective intervention to decrease stimulant use in adults [9]. The core principles of CM are to identify and target therapeutic behavior(s) (e.g., drug abstinence or decrease in use), specify a schedule with which to monitor the behavior(s), provide positive reinforcement via a tangible reward for achieving the behavior or a loss of privilege when the target behavior is not achieved. For adults with co-occurring Opioid use disorder (OUD) and Stimulant use disorder (StUD), CM reduces stimulant use for those receiving MOUD [9]. Though not studied as a treatment approach for adolescents with StUD, CM has proven to be an effective treatment for adolescent cannabis use disorder and retention rates for patients engaging in treatment [10].

There is an urgent need for new approaches to address rising adolescent overdose deaths and improve treatment of youth OUD, including those with co-occurring StUD. Combination Long-acting injectable buprenorphine (LAIB) and CM may be an important but underutilized treatment for youth under age 18 years. We present the first known case where an adolescent patient was engaged and retained in care using LAIB and CM to treat co-occurring opioid and stimulant use disorder.

## Case presentation

A 14-year-old presented to clinic after having a non-fatal overdose on fentanyl in December 2021 and was started on buprenorphine while inpatient at a local hospital. The patient was discharged to the only known outpatient primary care clinic in her county who was actively providing MOUD for adolescents. The patient had a significant history of SUD, reporting first use of alcohol and cannabis at age 9 y.o., and initiation of opioids at age 12 y.o. They reported using methamphetamine daily for 9 months and as many as five 30 mg (non-prescribed) oxycodone pills per day prior to their admission to the hospital. They have a history of mood disorder, anxiety and oppositional defiance disorder and were previously admitted to three different inpatient rehabilitation programs for their mental health and SUD diagnoses. They reported negative experiences at the programs and declined to enter another program to support their sobriety.

They established care at our clinic shortly after their hospital stay and was continued on sublingual buprenorphine. In addition, they and their parent were provided with naloxone kits and fentanyl test strips with nurse-led education about overdose prevention and harm reduction. The clinic also became their designated medical home. They were connected to a therapist, psychiatrist and a substance use counselor, through the local county health system. After hospital discharge, they were seen weekly to continue their treatment with sublingual buprenorphine. On the third week, they had an unexpected urine toxicology result for fentanyl and methamphetamine, and they reported using methamphetamines without knowledge of fentanyl contamination. At this point, they had been stable on 16 mg sublingual buprenorphine and had been adherent to the medication. Of note, they reported not feeling any physical effects of fentanyl, suggesting a possible protective benefit from the buprenorphine. At this time, we offered a monthly formulation of buprenorphine, LAIB, as they were reporting difficulty adhering to a daily medication and did not like the taste of the sublingual film. In collaboration with their care team and parent, the patient decided to switch to LAIB to ensure medication adherence. This is also when we began the discussion with the parent and patient about piloting a CM intervention as part of the treatment plan to address stimulant use. They and their parent agreed to weekly gift cards for adherence to regular weekly appointments and point of care urine toxicology testing with expected results. To avoid manipulation of the urine toxicology screen, we followed our clinic urine toxicology policy that includes a non-observed urine screen with the nurse collecting urine and having patient belongings left in the exam room. Upon initiation of the CM plan and LAIB, they remained engaged in care and had a period of abstinence for 4 months, something they and their father reported as their longest period of sobriety since age 12. Beginning in May of 2022 the patient began to have more unexpected urine toxicology results for methamphetamine. Between May 2022 and September 2022, they had a total of 6 (out of 14 visits) positive urine toxicology test resultsfor methamphetamine but no positive results for fentanyl. Given the increasing number of unexpected results, the treatment team and the parent decided to rethink the plan. At this point the parent gave the patient two options: (1) Another unexpected urine toxicology test would result in an admission to an inpatient treatment facility, (2) If they were able to maintain expected urine toxicology results at their weekly appointments, the parent would continue to give her the weekly gift cards and, over time would consider adopting a puppy. They maintained abstinence for the following 2 months and the puppy was adopted at the end of November. The patient continued to engage in care with us and her team at the intensive outpatient program. For the past 19 months they have remained engaged in primary care with 100% adherence with monthly appointments (for LAIB administration) and expected urine toxicology tests since 9/22/2022, 11 months total. At the most recent visit, they reported using her incentive money to purchase an electric bicycle. They have been able to do this all while living at home with their parent.

## Discussion

In this case we found the implementation of CM principles in addition to LAIB helped sustain patient engagement in care. In getting to know the patient and their father, it was evident that addressing co-occurring mental health disorders, engaging with the parent, and ensuring the patient was in school, were all contributing factors to their success. Treatment from their therapist, substance use counselor, and psychiatrist addressed the aforementioned factors, however, when asked, the patient said the rewards were the greatest motivator to sustain their abstinence. This treatment has led to the longest periods of sobriety for them, reduced her risk of overdose, and provided them with positive rewards for their engagement in care. In adults, CM is effective when it is being implemented but the effects wane when it is discontinued [11]. Since they have been engaged in care and have been stable, our plan with the patient is to continue with CM with longer periods in between visits if they are meeting their goals. In the beginning we met weekly, after 6 months we met biweekly, and now we are meeting monthly when they are due for LAIB administration. We will continue monthly LAIB administration for the next year and re-assess at that time. Among adult patients with OUD, treatment discontinuation or tapering off MOUD is associated with overdose [11]. Unfortunately, there is limited data to guide long-term treatment decisions for adolescents with OUD. For this patient with a significant history of serious consequences including overdose, it is reasonable to continue treatment with LAIB. However, it is unlikely that they will want to remain on LAIB for the rest of their life and the treatment team will need to have careful conversations with their parent about the risks and benefits of discontinuing medications. There is an urgent need for research to guide decisions about MOUD discontinuation for adolescents given the risk of overdose and adverse health outcomes if there is a recurrence of substance use.

The benefit of providing a medical home to offer outpatient addiction treatment for adolescents holds significant potential. For adolescents, who often have ambivalence about their use and may not want to be treated with MOUD indefinitely, they can remain engaged with a trusted treatment team. This primary care engagement ensures an infrastructure to allow for seamless re-initiation of MOUD or other behavioral treatment if desired by patient or indicated. Currently, fewer than 5% of adolescents in the United States with OUD are offered MOUD [12]. This case demonstrates how offering MOUD in primary care ensures low barrier, evidence-based treatment and is an important solution to addressing the staggering statistic. Maybe most important, there is an opportunity to demonstrate to adolescents that addiction treatment can be provided in their medical home, with compassion and lack of judgment, shifting the paradigm of treatment based on punitive approaches to one based on evidence and aligned with their goals for health.

## Data Availability

Not applicable.

## Declarations

### Article summary

Treating adolescents with opioid use disorder and stimulant use disorder with an evidence-based approach in a pediatric primary care clinic.

## Abbreviations

OUD: Opioid Use Disorder
CM: Contingency Management
MOUD: Medications for Opioid Use Disorder
LAIB: Long-Acting Injectable Buprenorphine
SUD: Substance Use Disorder
StUD: Stimulant Use Disorder
